# Performance evaluation of automated scoring for the descriptive similarity response task

**DOI:** 10.1038/s41598-024-56743-6

**Published:** 2024-03-14

**Authors:** Ryunosuke Oka, Takashi Kusumi, Akira Utsumi

**Affiliations:** 1 Mitsubishi Electric Corporation, Kamakura, Kanagawa 247-8501 Japan; 2 Graduate School of Education, Kyoto University, Yoshida-Honmachi, Sakyo-ku, Kyoto, 606-8501 Japan; 3 Graduate School of Informatics and Engineering, University of Electro-Communications, 1-5-1 Chofugaoka, Chofu-shi, Tokyo 182-8585 Japan

**Keywords:** Human behaviour, Information technology

## Abstract

We examined whether a machine-learning-based automated scoring system can mimic the human similarity task performance. We trained a bidirectional encoder representations from transformer-model based on the semantic similarity test (SST), which presented participants with a word pair and asked them to write about how the two concepts were similar. In Experiment 1, based on the fivefold cross validation, we showed the model trained on the combination of the responses (*N* = 1600) and classification criteria (which is the rubric of the SST; *N* = 616) scored the correct labels with 83% accuracy. In Experiment 2, using the test data obtained from different participants in different timing from Experiment 1, we showed the models trained on the responses alone and the combination of responses and classification criteria scored the correct labels in 80% accuracy. In addition, human–model scoring showed inter-rater reliability of 0.63, which was almost the same as that of human–human scoring (0.67 to 0.72). These results suggest that the machine learning model can reach human-level performance in scoring the Japanese version of the SST.

## Introduction

Crystallized intelligence is knowledge acquired from culture, education, and daily experiences^1^. Because crystallized intelligence is related to language production (e.g. sentence completion^2^) and comprehension (e.g. irony detection^3^) abilities, evaluating crystallized intelligence is important in both clinical and experimental settings. For example, in clinical setting, by using crystallized intelligence score subtracted by fluid intelligence (the ability to solve problems that cannot be solved using existing knowledge) score could be a predictor of preclinical Alzheimer’s disease^4^. For another example, in experimental setting, creativity score (as operationalized by the Finke Creative Invention task, which ask participants to generate something new but meaningful^5^) is related to the crystallized intelligence (overall correlation between the crystallized intelligence and the creativity score was *r* = 0.42^6^). Finally, the meta-analysis study showed crystallized intelligence predicted the real-world attainment (i.e. job performance) than fluid intelligence^7^ (see also Schmidt^8^) how the crystallized intelligence related to academic attainment, occupational performance, and mental function at older ages). Thus, crystallized intelligence is not just important as the language abilities but also important as preclinical diagnosis, creativity, and real-world attainment.

Crystallized intelligence is measured using three facets: vocabulary, information, and similarity. Among the three, the similarity facet, which measures participants’ ability to find the commonality of the two given concepts, is important because it is the only facet that requires not only the ability to retrieve knowledge but also the ability to evaluate the aptness of the response (i.e. participants have to choose the best commonality that captures the nature of the two given words).

More concretely, the similarity facet is measured using the descriptive similarity response task (in short, “similarity task”), which presents a word pair (e.g. “diamond – snowflake”) to participants and asks them to write about how the two concepts are similar (e.g. “has a crystal structure”). Participants’ responses are scored into three classes (2 points [pts]: perfectly captures the relationship; 1 pt: partially captures the relationship; and 0 pts: bad response) using classification criteria rubrics designed by psychological professionals. Though the rubrics are carefully designed for both each word-pair criteria (e.g. “diamond – snowflake” has three criteria for 2 pts and seven criteria for 1 pt) and overall instruction for scoring (e.g. responses based on participants personal experiences were scored 0 pts; for example, “the item reminded me of a scene from Indiana Jones” to “riddle – labyrinth”), the final scoring decision depends on the evaluators.

Although the similarity task is a useful measure to assess participants’ crystallized intelligence, evaluators (e.g. psychological professionals) spend a lot of time scoring participants’ responses. For example, based on the first author’s experience in the scoring similarity task, it took approximately two min to score each item. Therefore, if there are 20 items for the similarity task, it takes approximately 40 min for scoring, which is hard work. A tool to reduce evaluators’ scoring time would be helpful.

To reduce evaluator scoring time, applying an automated scoring system (e.g. machine learning) to a similarity task is a good candidate method. Here, we briefly explains how machine learning techniques can apply to the task like an automated scoring system. Machine learning is the statistical algorithm learns from data, extract patterns and solve the task. In this article, we focus only on the supervised learning, an approach in machine learning where a model learns from inputs and labelled outputs. There are five steps to complete the supervised learning. First, we needs to prepare the data. For example, in the similarity response task, there are word-pairs (e.g. “diamond” and “snowflake”) and the sentence to explain the similarity of given two concepts (“has a crystal structure”) as the inputs, and the points (e.g. 0, 1, and 2 pts) as the labelled outputs. Second, we needs to select the model which can correctly predict the expected results. In the similarity task, we needs to prepare the model which can transform the words-pair and sentence into points. Third, we needs to train the model so that it can produce the expected results even when presented with inputs that were not seen in the prepared data. Fourth, we needs to evaluate the model. This could be done by splitting the data into training data and test data (as we do in Experiment 1 in this article) and checking how the trained model correctly classifies the test data. Finally, we needs to use the model to predict unseen data (as we do in Experiment 2). Following this process, the supervised learning aims to create the model which can transform the input to the expected output using labelled data.

Machine learning approaches performed well in the short-answer scoring task, which focused on the content accuracy of participants’ responses to the rubrics. The similarity task is a type of short-answer scoring task; the similarity task is scored based on multiple classification criteria, and participants’ responses are mainly short compared to other types of classification tasks (e.g. document classification). For example, Riordan et al.^9^ showed that applying a machine learning-based scoring model (i.e. consisting of an embedding layer, convolutional neural networks, long short-term memory networks, and a linear classification layer for scoring) to a short-answer scoring task (i.e. SRA^10^, which consists of questions, reference responses, and each participant’s response; the question is derived from the student responses from interacting with a tutorial dialogue system) showed better performance than at baseline (non-machine learning approach). In addition, Mizumoto et al.^11^ proposed a bidirectional long short-term memory-based scoring model (i.e. consisting of an embedding layer, bidirectional long short-term memory layer, and sigmoid layer to classify each rubric, and summed all rubric scores to calculate holistic scores) and revealed that the model outperformed the support vector regression model^12^ and the model performance (average 86% accuracy) was comparable to human performance (average 87% accuracy). Both studies showed that by feeding approximately 1000 training data points, the model showed good performance and sometimes reached human-level performance in short-answer scoring tasks. Although these studies did not evaluate the model performance on the similarity task, by using this type of automated scoring system on the similarity task as a reference, evaluators can reduce scoring time.

The aim of this study was to test whether a machine learning-based automated scoring system can simulate human similarity task performance. If the system shows a comparable performance to that of humans, the model can help psychological professionals score clients’ crystallized intelligence more efficiently.

As our machine-learning-based automated scoring system, we chose a Bidirectional Encoder Representations from Transformers (BERT)-based model^13^. BERT is a stack of transformer layers^14^ capable of learning flexible representations from the training data. When applying supervised learning to sentences (i.e. the similarity response task), it is crucial to select a superior pre-trained language model that can accurately capture the semantics of words and sentences. BERT is one model of this line – in BERT, the vector of words and sentences can not only captures the left-to-right and right-to-left context of the word (as in word2vec^15^, which is previously one of the best language model) but also utilize the meaning of long context (by the help of transformer layers). As a result, BERT is succeeded to output high quality words and sentence vector. By using BERT as the language model, the model shows a strikingly high performance in language understanding, question answering, and common-sense inference^13^. We selected BERT for three reasons: First, the pre-trained BERT model, which learns word embedding from Wikipedia and other Internet documents worldwide, is easily implemented using the Hugging Face platform (e.g. https://huggingface.co/cl-tohoku/bert-base-Japanese). This advantage makes it easy to provide the code and model to replicate our results; the implementation becomes simpler compared to previous studies that applied complex models to short-answer scoring^9,11^. Second, because the pre-trained BERT model has already learned word and concept knowledge in the pre-training phase (i.e. whole-word masking and next-sentence prediction), we need only a small amount of training data to adjust the model to a certain task (e.g. similarity task). This “pretrain, then fine-tuning (so called transfer learning)” is common to use BERT model and showed strikingly high performance in language understanding, question answering, and common-sense inference^13^. Third, BERT can correctly understand the semantic similarity of words, similar to humans. A recent review showed that by feeding psychological data—feature norms, which are dozens of pairs of conceptual knowledge (e.g. a bird) and its features (e.g. has feathers, is fluffy^16,17^)—to BERT, the model can simulate human cognition regarding similarity perception^18^. For example, the BERT model reflected asymmetry perception of two-words similarity (human perception of similarity sometimes does not show symmetry; “age is an era” is not equal to “era is an age^19^”), distinguished similarities from synonymity (the model and human similarity judgment scores^20^ have Pearson correlations that ranged 47–0.51.) and reflected human-like within-category similarity judgment (the model and human similarity judgment scores had Pearson correlations that ranged 0.53–0.58^21^). Although previous studies did not determine whether the BERT model can simulate human similarity task performance because the model can capture human similarity perceptions (i.e. Likert rating of similarity by human judges^19,20^), there is a possibility that the model accurately mimics human performance even in similarity tasks.

As a human similarity task, we focused on the Japanese version of the Semantic Similarity Test^22^ (SST). The original SST^23^ is a test that mimics the composition of the similarity task in the Wechsler adult intelligent scale (WAIS; a de facto standard test of participants’ intelligence) but uses different word pairs and rubrics. The original SST is administered in English. There were two significant differences between the WAIS similarity task and the SST. Although the WAIS similarity task asks participants to respond orally, the SST asks participants to respond in writing. However, although the word pairs and rubrics for each were not publicly available in the WAIS similarity task, all the word pairs and rubrics for each were publicly available in the SST. The Japanese version of the SST follows the original SST: most word pairs and rubrics are the same as in the original SST, but some word pairs and rubrics are different based on the pilot study. The Japanese version of the SST was also administered in a written format. The Japanese version of the SST scores showed weak but positive correlations with the Japanese vocabulary size estimation test^24^ (*rs* = 0.29–0.31^16^), which estimated participants’ vocabulary size (strongly related to crystallized intelligence) based on a recognition test (i.e. participants’ tasks were to choose the words they knew as much as possible from a given word list). The results imply that the Japanese version of the SST has convergent validity for estimating participants’ crystallized intelligence.

To evaluate the model performance on the Japanese version of the SST, we prepared three training datasets: participant responses obtained in the pilot study (responses), classification criteria for each word pair of the Japanese version of the SST (classification criteria), and the combination of responses and classification criteria (combination). Previous studies^9,11^ showed by using trained participants’ responses, the automated scoring system displayed high performance (sometimes comparable to human performance) on short-answer scoring tasks. Thus, we expect that the BERT model trained by responses will show comparable performance to the human annotator (i.e. inter-rater consistency).

However, a model trained using classification criteria on an automated scoring system has remained unclear in previous studies. There were three differences between the responses and the classification criteria. First, although responses are sometimes strange (e.g. incomplete or nonsense responses), the classification criteria are written in a complete sentence and are clear because a professional created it. Second, the number of classification criteria was smaller than the number of responses. Third, the classification criteria do not have 0-pt criteria; this is because, in this study, we did not use overall instructions for scoring (e.g. responses based on participants’ personal experiences were scored 0 pts). To collect the 0-pt criteria of a word pair, the criteria of other pairs are taken at random (e.g. “bird – airplane”: “has crystal structure”). Although we do not have an a priori hypothesis, we anticipated the classification accuracy calculated from the model trained by classification criteria to be lower than that of the model trained by responses and combinations because the number of criteria is small, which would lower the model performance.

Two experiments were conducted. Experiment 1 examined whether the model trained by responses (response model), the model trained by classification criteria (classification criteria model), and the model trained by combination (combination model) trained by data collected in the pilot study^22^ could achieve human-level performance. In this experiment, the training data and test data were divided by fivefold cross validation manner: that is, 80% of the data are used for training and 20% of the data are used for test. We also examined whether the use of the same word pairs in the training and test data affected the model performance. In our dataset, each word pair has multiple responses and criteria. In model training, there is a possibility that whether the same word pair is included both in training data and test data (e.g. “bird – airplane” is included in both training data and test data) or not (e.g. “bird – airplane” is included in training data but test data) affects the model performance. To test this, we prepared both the word-pair condition and the without-word-pair condition and compared model performance. Experiment 2 examined whether the models showed human-level performance even in test data prepared from different participants (i.e. Study 1^22^). If the model can achieve human-level performance in Experiment 2, we can conclude that it can be applied to unseen responses from future participants.

## Experiment 1

### Methods

#### Design

To compare the effects of the dataset type (i.e. responses, classification criteria, and combinations) and whether the same word pairs were used in the training and test data, we set six conditions. First, in the “responses/same-pair” condition, both the training data and test data in each fold (i.e. 5) must include all word pairs (but remove the same response). Second, in the “responses/different-pair” condition, there was no overlap of word pairs in each fold between the training data and the test data: 4 to 5 word pairs were only included in the test data, and the rest ware only included in the training data. Third, in the “classification criteria/same-pair” condition, all the criteria were included in the training data and the test data of each fold were the same as that of the “responses/same-pair” condition. Fourth, in the “classification criteria/different-pair” condition, only the criteria that were derived from the same word pairs in each fold used in the training data of “responses/different-pair” condition were included in the training data. The test data in each fold were the same as in the “responses/different-pair” condition. Fifth, in the “combination/same-pair” condition, all the combination of responses and classification criteria (except duplication) were included in the training data. The test data of each fold were the same as that of the “responses/same-pair” condition. Finally, in the “combination/different-pair” condition, only the criteria that were derived from the same word pairs in each fold used in the training data of the “responses/different-pair” condition were included in the training data. The test data in each fold were the same as in the “responses/different-pair” condition.

#### Datasets

We used the responses, classification criteria, and combinations obtained by Oka et al. (under review)^22^ as datasets. The response dataset comprised 1600 responses from 80 participants (*M*_*age*_ = 42.4 years, 50 men) for 20 word pairs. Although the pilot study conducted by Oka et al. (under review) included 24 word pairs, because the purpose of this pilot study was to determine which items to include as word pairs of the Japanese version of the SST, and four items were dropped in the main analysis, we used the same 20 word pairs as a target in this study. All the data used in Experiment 1 and Experiment 2 were obtained from Oka et al. (under review)^22^. All participants provided written informed consent to these experiments. All researches conducted in Oka et al. (under review)^22^ followed the Declaration of Helsinki and approved by the Ethics Committee at the Graduate School of Education, Kyoto University.

Examples of word pairs, participant responses, and scores are summarized in Table 1. The sentences in participants’ responses were short. In addition, 0 pt responses included incomplete responses (e.g. in “time is like a flowing river,” no mention of what is like a river flow) and responses that deviated from common knowledge (e.g. a diamond is not “fragile”). To test the inter-rater reliability of the classification, the first and second authors individually scored 11% of participants’ answers. Fleiss’ kappa was calculated as an index of inter-rater consistency and found that the consistency was moderately high (Fleiss’ kappa = 0.67, *z* = 10.1, *p* < 0.001). Responses with different scores between authors were discussed and discrepancies were resolved. Subsequently, all the responses were scored again by the first author.

**Table 1:** An example of the word pairs, participants responses, and the scoring results (English translation with original Japanese text).

Word pair	Participant response	Score
Bird–airplane (鳥–飛行機)	Flies (空を飛ぶ)	2
Bird–airplane (鳥–飛行機)	Has wings (羽がある)	1
Time–river (時間–川)	Runs (流れる)	2
Time–river (時間–川)	Long and endless (長く果てしない)	1
Time–river (時間–川)	Time is like a flowing river (時は川の流れのように)	0
Diamond–snowflake (ダイヤモンド–雪片)	Crystal structure (結晶構造)	2
Diamond–snowflake (ダイヤモンド–雪片)	Sparkly stuff (キラキラしたもの)	1
Diamond–snowflake (ダイヤモンド–雪片)	Fragile (脆い)	0

The classification criteria included 413 criteria for 20 word pairs. For the classification criteria dataset, there were additional zero-pt classification criteria. These were collected as follows: in each word pair, we sampled half the number of criteria from the rest of the word pairs (i.e. we did not include correct word pair-criteria pairs). We then prepared the pseudo 0-pt labels by combining correct word pairs (e.g. “bird – airplane”) and sampled (incorrect) criteria (e.g. “bird – airplane” to “crystal structure” sampled from “diamond – snowflake”). As a result of combining the original classification criteria and pseudo 0-pt labels, the classification criteria dataset had 616 criteria. Examples of the word pairs, classification criteria (including pseudo-criteria for 0 pts), and scores are summarized in Table 2.

**Table 2:** An example of the word pairs, classification criteria, and the scores (English translation with original Japanese text).

Word pair	Classification criteria	Score
Sun–light bulb (太陽–電球)	Shed light (光を放つ)	2
Sun–light bulb (太陽–電球)	Heats up (熱を持つ)	1
Sun–light bulb (太陽–電球)	Fun (楽しい)	0
Thinking–net (思考–網)	Woven in (編み込まれている)	2
Thinking–net (思考–網)	Have an eye (目がある)	1
Thinking–net (思考–網)	Fast (速い)	0
Memory–prison (記憶–牢獄)	Be seized (捕われる)	2
Memory–prison (記憶–牢獄)	Packed (詰め込む)	1
Memory–prison (記憶–牢獄)	Harmonize (調和する)	0

Finally, the combination dataset comprises the response and classification criteria datasets. After eliminating duplicate items, the final combination dataset contained 1591 word pairs and items (response and classification criteria).

#### Models

To score participants’ responses automatically, we used a pre-trained BERT model (https://huggingface.co/cl-tohoku/bert-base-japanese) as the base model. The BERT model analysis was performed using Python 3.10.12. The Hugging Face library (transformer) version was 4.31.0, and the Torch version was set to 1.10.0. We used the BertForSequence Classification library to classify the responses into three classes (2 pts, 1 pt, and 0 pts). Because the Japanese version of SST is a classification task, the last layer of the BERT model was combined with a linear classification layer with cross-entropy loss. To fine tune the BERT model, the input of each model in the sixth condition was set to “[CLS]WORD1[SEP]WORD2[SEP]RESPONSE(or CLASSIFICATION CRITERIA).” Here, [CLS] and [SEP] are special tokens used to separate each feature (i.e. WORD1, WORD2, and RESPONSE/CLASSIFICATION CRITERIA). For example, if the word pair was “bird – airplane” and the response was “flies,” then the model input became “[CLS]bird[SEP]airplane[SEP]flies”. The output of each model for the six conditions was set as the score.

In addition, because the amount of data differs among the three classes (e.g. the number of 0 pts in the response dataset was extremely small relative to other scores because many participants could find at least some commonality between word pairs), when calculating the cross-entropy losses, we weighted the cross-entropy loss function based on the reciprocal of the number of each label divided by the total number of labels.

Here, we describe the specific parameters in detail. Regarding the training details, the batch size was set to 32 because when the number increased (e.g. 128), the model performance drastically dropped (a small number of items might have caused this). Early stopping, with patience times set to 3, was adopted. The number of epochs was set to 10. The learning rate for each model was set as 0.0002. The weight decay was set to 0.01. All other parameter settings followed the default settings (i.e. https://huggingface.co/docs/transformers/model_doc/bert).

### Results

The model performance is summarized in Table 3. There are four indices of model performance, and there were four measures (accuracy, precision, recall, f1 score). In addition, there are two ways to calculate the average (macro average and weighted average) for precision, recall, and f1-score. The mean precision, mean recall, and mean f1 scores were calculated using the average of fivefold performances. In all measures, “combination/same-pair” condition showed the highest performances (accuracy: 0.828, weighted average of mean precision: 0.828, weighted average of mean recall: 0.828; summarized in the fifth row in Table 3). These results show that the BERT model trained using a combination of responses and classification criteria was the best model. Regarding the average accuracy among the three datasets (i.e. the average of the same pair and different pair in each dataset condition), the combination (0.821) showed a higher performance than the responses (0.598) and classification criteria (0.589). In addition, the average accuracy of the same-pair in each dataset condition (0.727) showed a higher performance than the different-pair condition (0.611).

**Table 3:** Model performance in each measure (Experiment 1).

Condition	Accuracy	Macro average			Weighted average		
		Mean precision	Mean recall	Mean f1 score	Mean precision	Mean recall	Mean f1 score
Responses/same-pair	0.724	0.636	0.666	0.640	0.742	0.724	0.730
Responses/different-pair	0.472	0.454	0.468	0.404	0.510	0.472	0.476
Classification criteria/same-pair	0.630	0.498	0.486	0.486	0.668	0.630	0.644
Classification criteria/different-pair	0.548	0.466	0.460	0.438	0.644	0.548	0.580
Combination/same-pair	**0.828**	**0.714**	**0.692**	**0.694**	**0.828**	**0.828**	**0.828**
Combination/different-pair	0.814	0.698	0.690	0.690	0.818	0.814	0.814

The highest performances in each performance measure are provided in bold.

### Discussion

In Experiment 1, we examined whether the response, classification criteria, and combination models trained using the data collected in a pilot study^16^ could achieve human-level performance. The results showed that the BERT model trained using the combination of responses and classification criteria exhibited high performance (i.e. 0.828 in accuracy). The results suggest that by aggregating the responses and classification criteria, the model can achieve human (may be, like) performance in the similarity task.

As expected, the use of the same word pairs in the training and test data affected model performance. It suggested that by including the same word pair (e.g. “bird – airplane”: “can fly”), the model learn the essence of the similarities between two word pairs and can predict the degree of similarity in the same word pairs and responses which were not included in the training data (e.g. “bird – airplane”: “have wings”). Even if the model did not learn the same word pairs in its training data, the BERT model could learn and predict the correct labels with high accuracy if the model learned the combination (accuracy: 0.814). This suggests that the model can predict not only the same similarity task (e.g. the Japanese version of the SST) but also the unseen but the same form of the similarity task (e.g. WAIS).

In Experiment 2, we trained the BERT model on three datasets and examined whether the test data prepared from different participants could also be correctly predicted. In Experiment 1, participants in the response training data were the same as those in the test data; even if the word pairs were removed from each other (i.e. different-pair condition), some participants’ responses might be trained in model learning. Thus, the result obtained in Experiment 1 could be because the model learned the habits of participants’ responses; that is, the model cannot be applied to the responses obtained from different participants. To eliminate this possibility, in Experiment 2, we applied the model to responses that differed from those of the participants and the timing of data collection.

In Experiment 2, we calculated the inter-rater reliability of the predictions of each model and the first-author classification on the dataset. As explained in the Methods section for Experiments 1 and 2, Fleiss’ kappa, which is the index of the inter-rater reliability of the first and second author, ranged 0.67 to 0.72. If Fleiss’ kappa was calculated by first author scoring and the model prediction (i.e. human–model scoring) was comparable to that of Fleiss’ kappa obtained in the previous study (i.e. human–human scoring), it suggests that the model has the ability to score participants’ responses as accurately as humans in the Japanese version of the SST.

## Experiment 2

### Methods

#### Design and datasets

To compare the effects of the dataset type (i.e. responses, classification criteria, and combination), we set three conditions. First, in the “responses in Experiment 1″ condition, we trained all the responses obtained in the pilot study^22^ as training data (1600 responses). Second, in the “classification criteria” condition, we trained all the classification criteria with pseudo 0-pt labels obtained in the “classification criteria/same-pair” condition” in Experiment 1 (616 criteria). Finally, in the “combination” condition, we trained the combination of responses in Experiment 1 and the classification criteria condition (except duplicates).

Following the training data, we prepared test data from the responses obtained in Study 1 by Oka et al. (under review)^22^. The original data comprised 2000 responses from 100 participants (*M*_*age*_ = 41.8 years; 52 men) for 20 word pairs each. To test the inter-rater reliability of the classification criteria, the first and second authors individually scored 22% of participants’ answers. Fleiss’ kappa was calculated as an index of inter-rater consistency. The consistency was moderately high (Fleiss’ kappa = 0.72, *z* = 21.2, *p* < 0.001). Responses with different scores between authors were discussed and discrepancies were resolved. Subsequently, all the responses were scored again by the first author.

In addition, for the item the first and second author scored (i.e. 22% of participants’ answer), we asked another two annotators (both woman, 50 and 51 years old each, and the highest level of education is completion of vocational school and junior college) without practical experience in clinical psychology and assessment to score individually. By recruiting another annotators (especially, excepts authors) and check the inter-rater consistency (i.e. Fleiss’ kappa), we can evaluate whether the moderately high inter-rater consistency of the annotators were not just because the annotators have skilled in scoring (i.e. authors) but because the scoring based on the classification criteria were easy and have enough consistency. Two annotators were provided the classification criteria made by authors and some sample classification obtained in Experiment 1 (31 items). We then calculated Fleiss’s kappa based on their scores. Results showed that the both Fleiss’ kappa calculated by all four annotators (first author, second author, and two new annotators; 0.73, *z* = 52.6, *p* < 0.001) and by these two new annotators (0.74, *z* = 21.8, *p* < 0.001) showed moderately high consistency. Therefore, we concluded the annotation criteria is clear and human evaluation of the task has enough variability.

In Experiment 2, because there was a possibility to inflate the model performance if the test data included multiple responses about the same content (e.g. many participants responded “can fly” for “bird – airplane”), we excluded duplication and final test data included 1309 unique responses.

#### Model training

The model training settings (pre-trained model, model input/output, and calculation of cross-entropy loss) and their hyperparameters were the same as those in Experiment 1.

### Results

The model performance metrics are summarized in Table 4. Regarding accuracy and weighted average of mean recall, the responses in Experiment 1 showed the highest performance (accuracy: 0.800; weighted average of mean recall: 0.800). In the weighted average of mean precision, the combination condition showed the highest performance (0.780). These results show that the BERT model trained using the responses in Experiment 1 and the combination of responses and classification criteria performed better than the classification criteria model.

**Table 4:** Model performance in each measure (Experiment 2).

Condition	Accuracy	Macro average			Weighted average		
		Mean precision	Mean recall	Mean f1 score	Mean precision	Mean recall	Mean f1 score
Responses in Experiment 1	**0.800**	**0.690**	0.620	0.620	0.770	**0.800**	**0.780**
Classification criteria	0.570	0.510	0.520	0.500	0.640	0.570	0.600
Combination	0.790	0.670	**0.650**	**0.660**	**0.780**	0.790	**0.780**

The highest performances in each performance measure are provided in bold.

Subsequently, we calculated the Fleiss kappa value for each model. The results showed that although the classification criteria model (0.312) showed poor performance, Fleiss’ kappa of the responses model (0.633) and combination model (0.627) showed high performance comparable to that of human–human scoring (0.67 to 0.72). In addition, to clarify the error pattern of the best model (response model), we prepared a confusion matrix that summarized the human labels obtained in the first author scoring in Oka et al. (under review)^22^ and the predicted labels obtained in the response model (Table 5). Although the model showed high performance in 2-pt (precision: 0.83, recall: 0.88, f1 score: 0.85) and 1-pt (precision: 0.78, recall: 0.85, f1 score: 0.82) labels compared with humans, the model showed low performance on 0-pt labels (precision: 0.47, recall: 0.12, f1 score: 0.19).

**Table 5:** Confusion matrix of the human label and predicted label obtained in responses in Experiment 1 model.

Model prediction	Human label		
	0 pts	1 pt	2 pts
0 pts	14	10	6
1 pt	87	538	61
2 pts	19	82	492

For detailed analysis, based on the confusion matrix of the responses in Experiment 1, we analyzed the errors for each human label. In the error of the 0 pt human labels, there were three types of misclassifications. First, some responses were classified as zero points based on the overall instructions for scoring, which were not included in our training data. For example, the responses based on phonological similarity (e.g. “marriage – alloy” to “phonologically similar (in Japanese, 結婚(ke-kkon) and 合金(go-kin) is similar)”; responses that just noted the phonological similarity were classified 0 pts) and the incomplete responses (e.g. “bird – airplane” to “air”; responses that were classified 0 pts) were defined 0 pt in overall instruction for scoring. Second, there were “there is no similarity” responses, and these were misclassified. Third, there were some responses in which there was no concrete similarity (e.g. “memory – prison” to “brain”). In the error of 1 pt human labels, although there were a few misclassification characteristics, 82 out of 92 misclassified responses were labeled as 2 pts. Finally, there were two types of misclassifications in the error of 2-pt human labels. One concerns the orthographic differences between the training and test data. For example, though “sun – lightbulb” to “light (in Japanese, 光る(hika-ru))” was included in training data, the same but different response “sun—lightbulb” to “light (in Japanese, ひかる(hika-ru)” in test data was misclassified as 0 pts. The other is that some 2-pt answers were included in the response but misclassified because there was 1-pt criterion. The overall instruction for scoring has a rule that if the 1-pt individual criterion is met, but the 2-pts individual criterion is also met, it is considered 2 pts. For example, “sword – pistol” to “where it is a tool to attack people (in Japanese, 人を攻撃する道具である),” tool to attack (攻撃する道具) should be scored 2 pts but misclassified into 1 pt (because only attack (攻撃) was classified into 1 pt in criteria).

**Figure 1:**
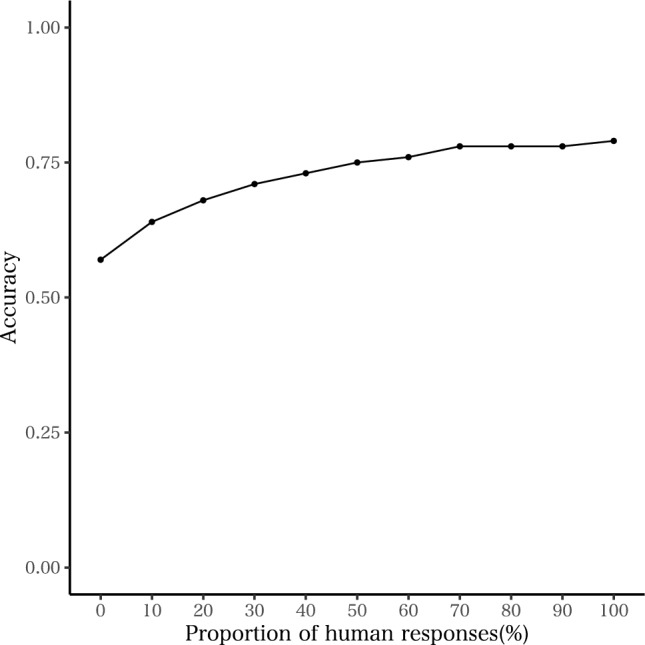
The proportion of human responses and the accuracy of the model.

Finally, to confirm the effect of the number of data, we trained the BERT model with the data combined by rubric data used in Experiment 1 and 2 (616 criteria) and randomly selected human responses data used in Experiment 1 (total: 1,219 responses). Because the model performance in “Classification criteria” condition showed low performance (i.e. 0.570 in accuracy) and “Combination” condition showed high performance (0.790), there was benefit of adding some annotated human responses in addition to the classification criteria. Therefore, it was useful to evaluate how many other human responses were needed to elevated the model performance other than classification criteria. To do so, the proportion of the selected data were increased by 10% increments from 0% (which was equal to the results obtained in “Classification criteria” condition) to 100% (which was equal to the results obtained in “combination” condition). The relationship between the proportion of human responses and the accuracy of the model was summarized in Fig. 1. As the line graph showed, with 50% (about 600) additional human responses made the model performance 75%. However, even in 70% to 100%, there were slight increment of the model performance. Therefore, though the performance will be increased if there are many more data to the combination model, we confirmed 600 additional data will be enough to reach the similar performance obtained in the full combination model.

### Discussion

In Experiment 2, we examined whether the test data prepared by different participants could also be correctly scored. The results revealed that the responses in Experiment 1 were scored with 80% accuracy. This result was the same as the best performance in Experiment 1 (83% accuracy in the combination/same-pair condition). Moreover, the combination model still exhibited high performance (i.e. 79% accuracy). These results suggest that the model trained on the responses (with 1,600 training data points) showed high performance in scoring the responses obtained from different participants. It also suggested that the results obtained in Experiment 1 were not owing to the model learning participants’ response habits but because the model learned the response similarity to word pairs.

In addition, based on the Fleiss’ kappa obtained in each model prediction and human scoring (i.e. the first-author classification in Oka et al. (under review)^22^ Study 1), we found that responses in the Experiment 1 model and combination model showed comparable results to those of human–human scoring in Oka et al. (under review)^22^. This suggests that by using the BERT model trained on the responses and the combination of the responses and classification criteria, the model could help raters (i.e. psychological professionals) by suggesting model prediction.

Moreover, the error analysis revealed that some misclassifications were caused by the lack of training data for the overall scoring instruction. This was especially true for the 0 pt label (because there were no individual criteria for each word pair).

Finally, we confirmed that if there were 600 more human responses with classification criteria, the model performance could reach the combination model performance. This could be the benchmark especially when the practitioner wants to make model performance better with classification criteria.

### Presentation Information

Part of this study (analysis of Experiment 1 without 5 fold cross validation) was presented at the 37th Annual Conference of the Japanese Society for Artificial Intelligence, Kumamoto-jo Hall, Kumamoto, Japan, June 6–9, 2023.

## General discussion

We tested whether a machine-learning-based automated scoring system could mimic human similarity task performance. Both Experiment 1 and Experiment 2 showed that by training the model on responses (approximately 1600 responses) and on the combination of responses and classification criteria, the model performance reached approximately 80% accuracy (the best performance in Experiment 1: 83%, Experiment 2: 80%). In Experiment 2, we confirmed that the inter-rater reliability between human and machine raters (Fleiss kappa: 0.63) was comparable to that between human raters (Fleiss kappa: 0.67–0.72). These results suggest that the machine learning-based model can achieve human-level performance when scoring the Japanese version of the SST.

Moreover, in Experiment 1, although the model performance decreased slightly, the model of the combination/without the word-pair condition showed high performance (81% accuracy) even if the training data did not include the word pairs used in the test data. This also suggests that the model has the potential to correctly score not only the same task (Japanese version of the SST) but also other similarity tasks with different word pairs (e.g. the WAIS similarity task).

As implied by some previous studies^18,21^ the BERT model trained on a similarity task exhibited good performance. Although previous studies did not examine the similarity task (i.e. participants were given word pairs and asked to respond to the commonality of these) but examined the similarity rating task^19,20^, we confirmed that the model was also applicable to the similarity task. Regarding language understanding, question answering, and common-sense inference^11^, the BERT model has strong capabilities to learn task representations to find similarities between word pairs. In addition, similar to the short-answer scoring task^9,11^, the BERT model can simulate human-level performance in a similarity task with approximately 1000 training data points (in this study, 1600). The model required only 80 participants for 20 word pairs. The size of the dataset is quite small compared to other language tasks (e.g. SQuAD 2.0^25^, which is a common benchmark in BERT and contains 100,000 question-and-answer pairs). This suggests that the similarity task requires relatively few training data for correct scoring.

The BERT model trained on the similarity task reduces the scoring time of evaluators by showing suggestions on which score to assign to participants’ responses. Although we did not calculate the time evaluators spent on scoring, including with and without the model, because the model provides the reference score (with approximately 80% confidence), their time to check the obvious items (e.g. frequently appearing responses) will decrease. Future studies should conduct experiments to verify whether the model actually helps with scoring.

There were at least two limitations for this study. For one thing, it was important to note that the automated scoring method utilizing language model (e.g. BERT) has some potential bias which will decrease the scoring performance of the response similarity task. For example, because the model we used (https://huggingface.co/cl-tohoku/bert-base-japanese) was trained only on Wikipedia, the model performance will be degraded if there were no document about the word-pair or the participants responses. Moreover, it was reported that some document-intrinsic bias (e.g. gender bias) occurred in Japanese Wikipedia. Panatchakorn et al.^26^ showed that BERT model trained on Japanese Wikipedia showed gender bias, which is examined by utilizing the revised version of Natural Language Inference (in short, NLI) task. NLI task is to judge whether the first sentence contradict/neutral/entailment to the second sentence. For example, if the first sentence is “the man played the tennis” and the second sentence is “the man used the racket and the ball”, the correct answer is “entailment”. In Panatchakorn et al.^26^, they mixed the gender-biased sentence as NLI task – that is, though two sentences were neutral (e.g. “the doctor played the tennis” and “the man played the tennis”), the subject words were biased (i.e. “many doctors are the man”). As expected, the BERT model trained on Wikipedia showed gender bias in this experiment- thus, even if the mixed sentence were designed to be “neutral”, about 40% of the sentences were misclassified into “contradict” and “entailment”. Though we do not include gender-related word pairs in the response similarity task, it is important to supposed there are documents related biases in the automated scoring system.

For the other thing, though the rubric is supposed to be the core of training set, we confirmed that learning the rubric (“Classification criteria” condition) showed worse accuracies (“Classification criteria/same-pair” condition in Experiment 1:0.630, “Classification criteria” condition in Experiment 2: 0.570) than those of “Combination” conditions (“Combination/same-pair” condition in Experiment 1: 0.828, “Combination” condition in Experiment 2: 0.790). We supposed there were three reasons. First, as the original rubric of SST^23^ and WAIS similarity subscale, it was difficult to cover all the possible responses beforehand in Japanese version of Semantic Similarity Test. Second, there were no 0 pt labels (except pseudo 0 pt labels we assigned) in classification criteria condition. Third, the classification criteria contains only a small number of examples (*N* = 616). One countermeasure for this issue is to add some human annotated responses to the classification criteria, as reported in results of Experiment 2. If we add 600 more data (in Japanese version of SST, about 30 participants data), the model performance can achieve the enough performance.

Finally, three promising areas for future studies should be addressed. First, future studies should handle the overall instructions for scoring the training data. In this study, we did not include the overall instructions for scoring in the model training. However, some tasks may be handled through preprocessing. For example, orthographic differences (e.g. kanji and hiragana in Japanese) are normalized by lemmatizing using a tokenizer (e.g. MeCab^27^ and Sudachi^28^). For another example, frequently generated incorrect responses, which can apply to many word pairs (e.g. “there is no similarity” responses), will be used for training data by combining these items to each word pair (e.g. “bird – airplane” to “there is no similarity” is scored 0 pts). Thus, preprocessing can be easily applied. Further, it is more challenging. For example, how can we define the model to correctly distinguish the response that did not include incomplete similarity but had a partial similarity (1 pt) from the response that included complete similarity but also had a partial similarity (2 pts)? One promising way to improve the model is to create NA categories other than 0/1/2 pt(s). For example, in current dataset, we assigned 2 pts for “where it is a tool to attack people” on “sword—pistol” even though the responses included two different criteria whose assigned scores are different (i.e. “attack” is 1 pt and “tool to attack people” is 2 pts). If we reannotated the dataset and assign NA to this kind of responses and again trains the model, the classification accuracy of the model would be improved because the confusing items are categorized into NA. These challenging tasks are future tasks for natural language processing.

Second, applying a GPT-based model (e.g. ChatGPT^29^) using zero-shot learning (e.g. by prompting) to the Japanese version of the SST will be important to compare whether the performances are comparable or overwhelming in this study. With the release of ChatGPT, researchers realized that the API could solve many types of semantic tasks. This was also true for similarity tasks. A recent study that compared ChatGPT performance and fine-tuned BERT model performance showed^30^ that the fine-tuned BERT model outperformed ChatGPT in a similarity task (e.g. STS-B^31^). Thus, we believe that the fine-tuned model trained on the Japanese version of the SST might show comparable (or outperform) performance to ChatGPT. Future studies should directly compare the performance of the model provided in this study and ChatGPT on the Japanese version of the SST. It would also be interesting to determine the best ChatGPT prompt for the similarity task.

Third, future studies should apply the BERT model to other WAIS tasks. WAIS tasks cover all three facets of crystallized intelligence (i.e. vocabulary, information, and similarity), and some tasks have a similar pattern to similarity: participants were asked to respond correctly based on oral responses. Thus, the transcripts of the response and item are easily collected and used for fine-tuning large language models (e.g. BERT). Can the model correctly score participants’ responses by collecting data from participants based on the WAIS or similar measurements? This question should be addressed in future studies.

In conclusion, this study broadens the scope of machine-learning-based automated scoring systems for similarity tasks using the fine-tuned BERT. Our results showed that the model scored with approximately 80% accuracy, which was almost the same as that of human inter-rater reliability. Future studies should apply this model to a more general crystallized intelligence test (e.g. the WAIS).

## Data Availability

The datasets generated and/or analysed during the current study are available from https://github.com/okaexp/Automated_scoring_of_SST.
